# BCAR: a fast and indel-tolerant barcode-sequence mapper

**DOI:** 10.1093/bioinformatics/btag557

**Published:** 2026-07-25

**Authors:** Bryan Andrews, Rama Ranganathan

**Affiliations:** Department of Biochemistry and Molecular Biology, The University of Chicago, Chicago, IL 60637, United States; Center for Physics of Evolving Systems, The University of Chicago, Chicago, IL 60637, United States; Department of Biochemistry and Molecular Biology, The University of Chicago, Chicago, IL 60637, United States; Center for Physics of Evolving Systems, The University of Chicago, Chicago, IL 60637, United States; Pritzker School of Molecular Engineering, The University of Chicago, Chicago, IL 60637, United States

## Abstract

**Motivation:**

DNA barcodes are commonly used as a tool to distinguish genuine mutations from sequencing errors in sequencing-based assays. In the presence of indel errors, utilizing barcodes requires accurate alignment of the raw reads to distinguish genuine indels from indel errors. Existing strategies to do this generally rely on aligners built for homology comparison and do not fully utilize quality scores. We reasoned that developing an aligner purpose-built for error correction could yield higher quality barcode-sequence maps.

**Results:**

Here, we present BCAR, a fast barcode-sequence mapper for correcting sequencing errors. BCAR considers all of the evidence for each base call at each position both during alignment and during final consensus generation. BCAR creates high-accuracy barcode-sequence maps from simulated reads across a broad range of error rates and read lengths, outperforming existing methods. We apply BCAR to two experimental datasets, where it generates high-quality barcode-sequence maps.

**Availability and implementation:**

BCAR source code, documentation, and test data are available from: https://github.com/dry-brews/BCAR or https://doi.org/10.5281/zenodo.20088816

## 1 Introduction

In high-throughput sequence-function mapping, DNA barcodes are commonly appended to libraries of genetic variants. A barcode is a short non-coding sequence placed on the same physical piece of DNA as a variant of interest. Generally, sequencing is performed in two phases. First, the entire piece of DNA is sequenced to map the barcode to the putatively functional DNA variant. Second, the barcode region is deeply sequenced to measure the effect of the variant, e.g. by measuring the frequency change of each barcode over the course of selection (Starita *et al*. 2018, Jones *et al*. 2020, Andrews and Fields 2021, Adamovich *et al*. 2022, Popp *et al*. 2025). Measuring the frequency of barcodes rather than the frequency of mutations offers a major advantage: without barcodes, one cannot distinguish between a sequencing error and a bona fide mutation, but barcodes make this distinction possible. During mapping, multiple reads are collected for each barcode, with genuine mutations occurring consistently on all reads and sequencing errors occurring sporadically.

**Figure 1:**
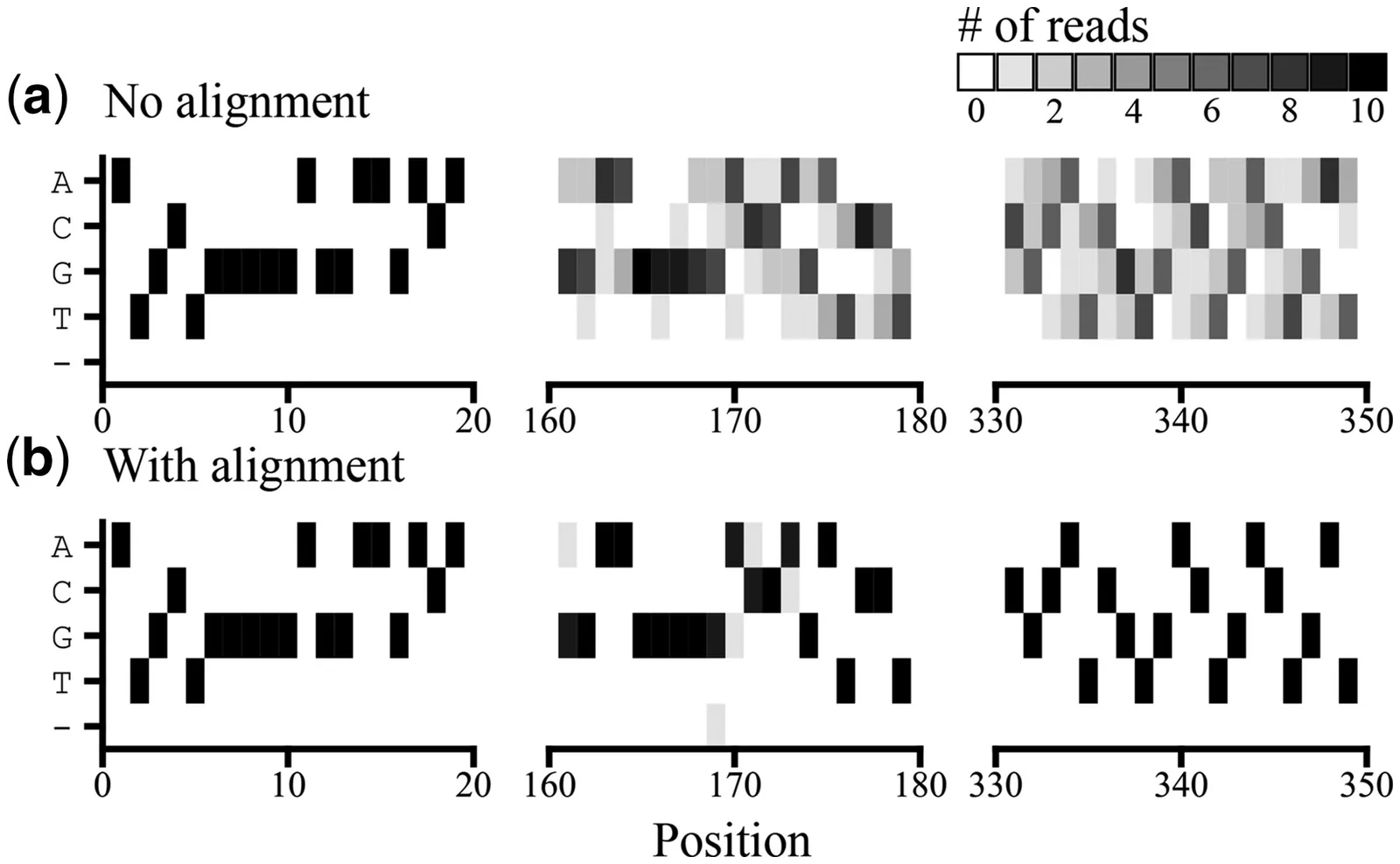
Position-weight matrices for all 10 raw reads associated with a particular barcode from an AVITI run of 16 million barcoded PDZ3 variants. (a) Base calls were counted at each position for all reads after adapter trimming, without alignment at the beginning (*left*), middle (*center*), and end (*right*) of the read. (b) As above, except the reads were aligned using Clustal Omega with default parameters (Sievers and Higgins 2018).

However, creating an accurate barcode-sequence map can be non-trivial when sequencing errors are common relative to bona fide mutations. *Indel* errors, where the read has a missing or extra base relative to the true sequence, are especially problematic. Once two reads fall out-of-phase, they will tend to disagree about every base call downstream of the indel (Fig. 1). Existing strategies address indels by using off-the-shelf multiple sequence alignment algorithms to align reads to a reference (e.g. alignparse (Crawford and Bloom 2019)) or to each other (e.g. PacRAT (Yeh *et al*. 2022)). Then, reads with indels are filtered out (alignparse) or heuristics are used to pick which read to trust (PacRAT).

Pragmatically, using filtering or heuristics to resolve indels can limit the broad applicability of an analysis tool. Filtering will tend to fail when the error rate is large relative to 1/L, where *L* is the read length, because most reads will have errors. Modern long-read sequencers can produce reads >10 kb, making filtering impractical even with low error rates. Heuristics, while useful, can be highly platform-dependent, with PacRAT being explicitly optimized for Pacific Biosciences reads.

On a more principled level, commonly used multiple sequence aligners are designed for comparing homologous sequences and are not necessarily well-suited for error correction. Sequencing reads generally have associated quality scores that estimate the per-base error rate, whereas most aligners have no notion of uncertainty about a base identity. Furthermore, most aligners assume phylogeny, using a guide tree to direct alignment, but phylogeny does not apply to sequencing datasets.

Therefore, we sought to build a barcode-sequence mapper that was explicitly designed to align sequencing reads containing errors. We supposed that a well-designed mapper should treat raw reads as collections of evidence about the true sequence, rather than as sequences per se, and that the mapper should handle uncertainty explicitly throughout the alignment and consensus generation process. In principle, this framework would allow us to generate accurate consensus sequences even when every read contains many errors, e.g. because the error rate is very high or because the reads are very long.

## 2 Methods

We present BCAR: Barcode Collapse by Aligning Reads. BCAR’s purpose is to quickly align the sequencing reads associated with each barcode in a dataset and generate a maximum-likelihood consensus sequence, including confidence scores for each base call. BCAR diverges from existing strategies by considering reads as matrices that contain the evidence for each base call at each position, rather than as strings of putative bases. The overall workflow consists of three phases:

In the first phase, BCAR extracts barcodes from the raw reads, counts them, and clusters barcodes that are within a user-configurable substitution and/or indel distance from each other using a greedy centroid clustering approach (MacKay 2003, Zorita *et al*. 2015) (Supplementary Material, section 2.1, available as supplementary data at *Bioinformatics* online). In the second phase, BCAR sorts chunks of raw reads on the basis of their barcodes so that all the reads associated with each barcode can be read at the same time, without reading the whole dataset into memory (Supplementary Material, section 2.2, available as supplementary data at *Bioinformatics* online).

**Figure 2:**
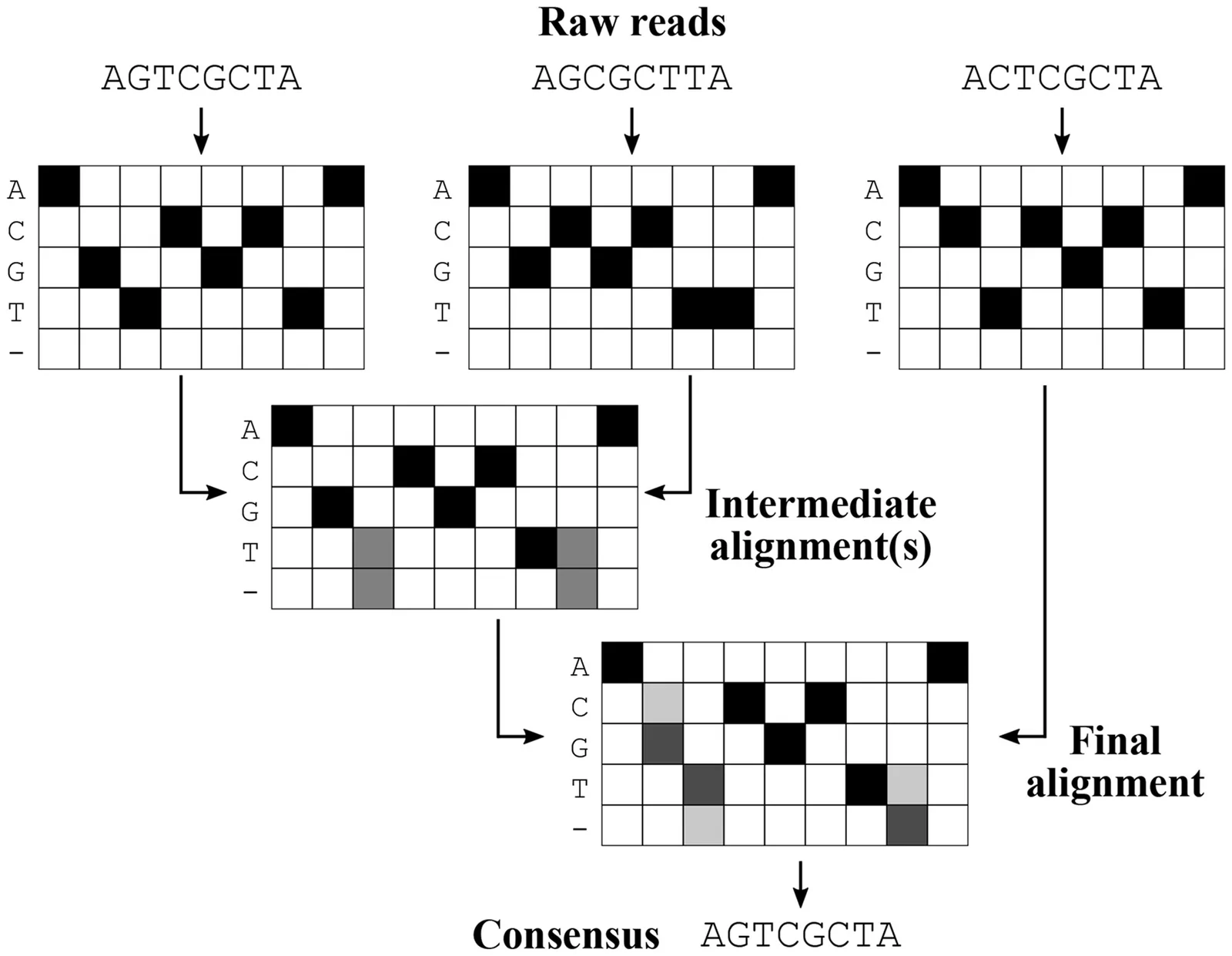
Flowchart with a toy model showing the progressive alignment strategy of BCAR. Reads are represented as arrays with entries corresponding to quality scores, then progressively aligned using a modified implementation of the Needleman-Wunsch algorithm (Needleman and Wunsch 1970). At each step, the new read is aligned against the current consensus, using a scaled cosine similarity to determine match scores between ambiguous positions. Gaps are removed from the final alignment during consensus generation.

In the third phase, the reads associated with each barcode are merged into a single consensus sequence. This processing can be parallelized across many compute threads, with each thread handling a single barcode at a time. The process is depicted in Fig. 2. Raw reads are converted to arrays where each entry represents the evidence for a base call at a given position. The arrays are globally aligned with each other, allowing for fractional match/mismatch scores when the identity of a position is ambiguous (Supplementary Material, section 2.4, available as supplementary data at *Bioinformatics* online). The evidence from the aligned positions is combined, and gaps may be introduced into the combined array. This process is then repeated until all of the reads corresponding to that barcode are accounted for.

At the end of the third phase, the final consensus array is converted back into a consensus sequence (Supplementary Material, section 2.6, available as supplementary data at *Bioinformatics* online). At each position, the base call with the most evidence is picked, and a quality score is calculated by using Bayes’ theorem to weigh the evidence for each of the potential base calls.

### 2.1 Handling barcode misassignment

BCAR has the primary role of correcting random indel and substitution errors that occur during sequencing. However, another category of errors arises when a barcode is misassigned to a set of mutations, which may occur by three mechanisms.

First, “index hopping” or similar phenomena may cause base calls arising from two molecules to be inappropriately mapped to a single read (van der Valk *et al*. 2020). When rare, disagreement between individual reads and the consensus caused by index hopping is well-handled by the core BCAR algorithm. However, if index-hopping is very prevalent, users may wish to filter consensus reads to a minimum number of raw reads. BCAR reports the number of raw reads associated with each barcode for this purpose.

Second, an error in the barcode portion of the read may cause two reads that represent the same true barcode to be mapped to different barcodes. As described above, BCAR allows clustering of barcodes within a user-specified edit-distance, using a greedy centroid clustering approach (Supplementary Material, section 2.1, available as supplementary data at *Bioinformatics* online). Rather than choosing a representative barcode for each cluster, each cluster is assigned an identification number. The consensus barcode for each cluster is determined after the fact from the consensus sequence and reported in the header for that consensus. This approach leverages the full power of BCAR to determine the “true” barcode sequence for each cluster.

Third, identical barcodes may be physically associated with different variants during library generation. This problem violates the assumption of random errors underlying BCAR’s implementation of Bayes’ theorem. As an example, a barcode with 100 reads, where 70 reads have base call “A” and 30 reads have base call “C,” would be confidently reported as “A” despite the extreme unlikelihood of having 30 independent errors agreeing on “C.” To address this, BCAR calculates a maximum minor allele frequency for each barcode group, which is reported in the header of each consensus read. The user can then filter against reads that have a minor allele frequency that they consider implausibly high.

## 3 Results

### 3.1 Accuracy on simulated reads

**Figure 3:**
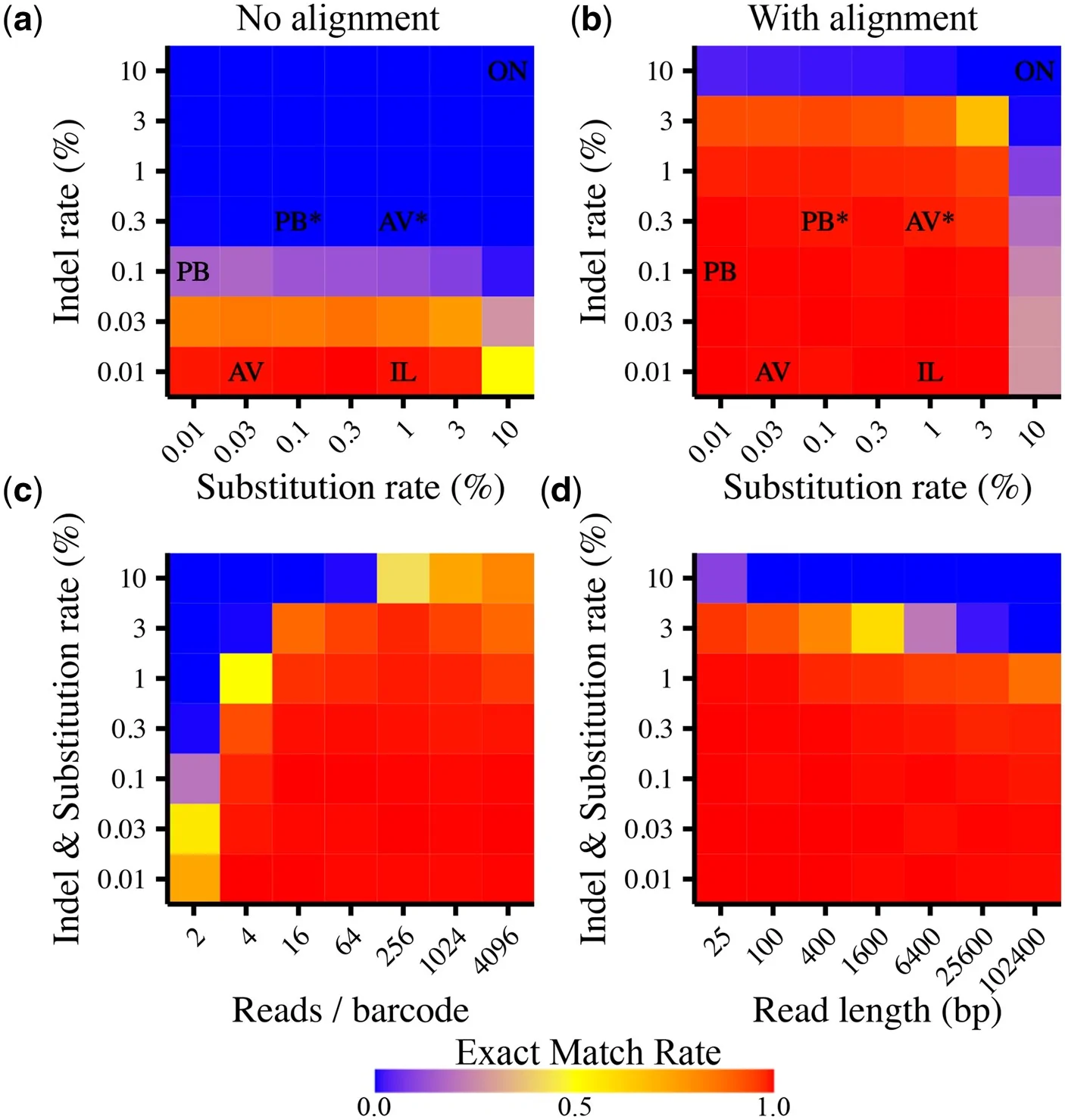
Reconstruction accuracy of BCAR on simulated reads. (a,b) Rate of reconstructing an exact match to the true sequence as a function of indel and substitution rates. Measured indel and substitution rates of commercial platforms. IL: Illumina MiSeq, estimated 0.85% substitution, 0.00635% indel error rate (Schirmer *et al*. 2015). PB: PacBio HiFi, estimated 0.008% substitution rate, 0.01%–0.21% indel rate (Wenger *et al*. 2019). PB*: PacBio HiFi, estimated 0.06% substitution, 0.38% indel error rate (this study). ON: Oxford Nanopore, estimated 10.2% substitution, 7.7% indel error rate (Leggett *et al*. 2016). AV: Element AVITI, estimated 0.05% substitution, 0.0075% indel error rate (Liu *et al*. 2024). AV*: Element AVITI, estimated 0.77% substitution, 0.39% indel error rate (this study). In (a), BCAR is prevented from aligning reads while in (b), alignment is performed with default parameters. Most platforms have high enough indel rates to benefit from alignment. (c) Reconstruction accuracy as a function of error rate and reads per barcode. Having more reads is always better, but the additional benefit of more reads is mostly saturated after ∼10 reads. (d) Reconstruction accuracy as a function of read length. Reconstruction accuracy declines somewhat with increasing read length when error rates are very high, but with lower error rates, long reads can be reconstructed with high accuracy.

Because real sequencing reads do not provide a ground truth to distinguish sequencing errors from bona fide mutations, BCAR’s accuracy was tested on simulated reads with defined rates of substitution and indel errors. BCAR perfectly reconstructs the true sequences from simulated reads across a broad set of indel and substitution rates (Fig. 3b), encompassing the measured error rates of most major sequencing platforms (Schirmer *et al*. 2015, Leggett *et al*. 2016, Wenger *et al*. 2019, Liu *et al*. 2024). In the absence of alignment (Fig. 3a), reconstruction fails except when indels are very rare, as in Illumina reads. BCAR benefits from having more reads per barcode, but most of the benefit comes from the first ∼10 reads, and accuracy gains with more reads are modest (Fig. 3c). BCAR functions well across a broad set of realistic read lengths. It is generally easier to reconstruct shorter reads, but BCAR performance decays much more slowly than 1/L, as demonstrated by successful error correction on >100 kb reads containing hundreds to thousands of errors on average (Fig. 3d).

### 3.2 Application to experimental data

**Figure 4:**
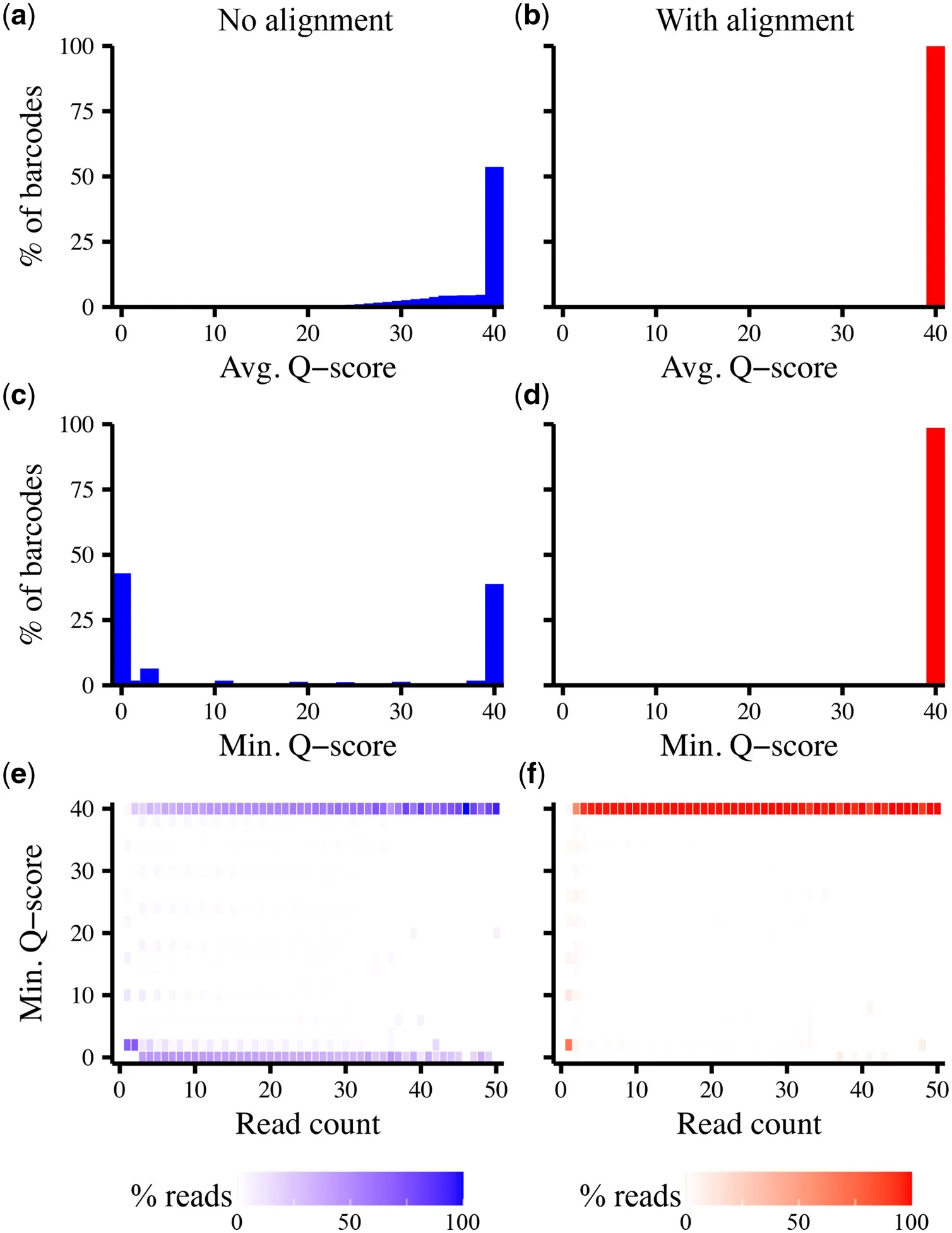
BCAR processing of PacBio HiFi reads from an experimental dataset. (a,b) Histograms of the average quality score of consensus sequences built from five or more reads, without (a) or with (b) alignment. (c,d) Histograms of the minimum quality score of consensus sequences built from five or more reads, without (c) or with (d) alignment. (e,f) Heat maps showing the minimum quality score of reads as a function of read count, without (e) or with (f) alignment. Each cell is shaded according to the percent of reads at a given read count that have the indicated minimum quality score.

To assess the value of BCAR on real data, we used BCAR to construct barcode-sequence maps for two experimental datasets. The first dataset consisted of a library of 7 40 000 barcoded variants of the KaiABC operon, for which we obtained 7.7 million reads of ∼3.5 kb using PacBio HiFi sequencing. Using BCAR, we estimate a 0.38% indel rate per position, meaning that the average read contains >10 indels. As shown in Fig. 4, alignment is critical to obtain a high-quality consensus sequence, because when BCAR is run in no-alignment mode, the majority of consensus sequences contain low-quality base calls (Fig. 4c), which are almost entirely fixed when alignment is performed (Fig. 4d). The second dataset consisted of a library of 19 million barcoded variants of the PDZ3 domain, for which we obtained 385 million reads of ∼350bp using Element AVITI sequencing. In this case, the benefit of alignment is less pronounced (Fig. S2, available as supplementary data at *Bioinformatics* online), but alignment still recovers a substantial number of high-quality consensus sequences that would otherwise be low-quality.

### 3.3 Comparison with existing methods

Recent progress has been made to develop tools for barcode-sequence mapping and UMI consensus generation. Existing software packages include fgbio (Fennell and Homer), PacRAT (Yeh *et al*. 2022), and alignparse (Crawford and Bloom 2019). For fgbio, no alignment is performed, and evidence is combined across reads using a Bayesian strategy similar to BCAR. For PacRAT, the reads associated with each barcode are aligned using MUSCLE (Edgar 2004), which does not utilize quality scores. Then, for positions with ambiguity, the base call from the highest-quality read is accepted as the consensus. For alignparse, the reads are pairwise aligned to a reference sequence using minimap2 (Li 2018), and reads with indels relative to the reference are discarded.

**Figure 5:**
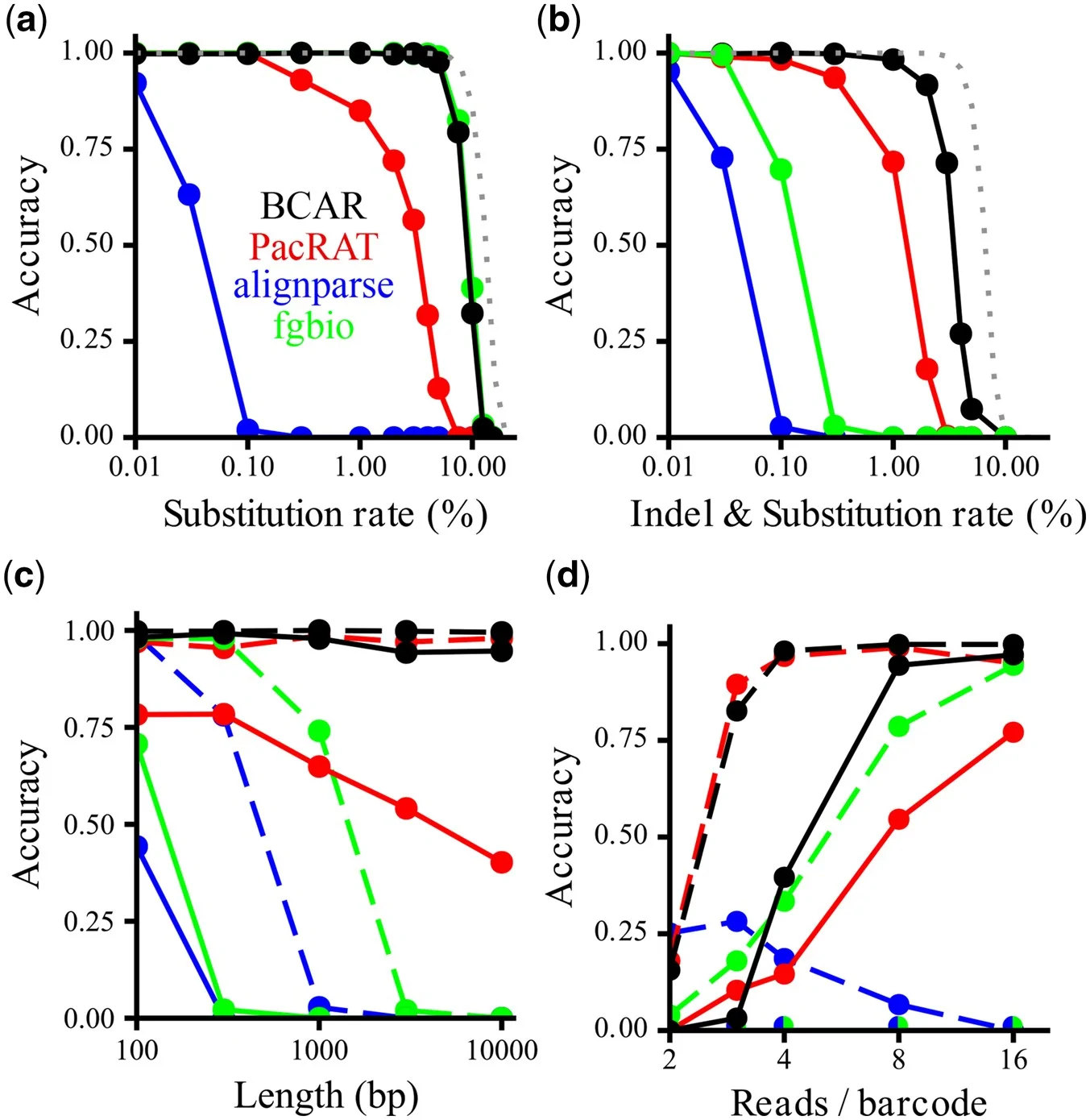
Comparison of BCAR’s accuracy on simulated reads to other methods. Accuracy, the fraction of true sequences reconstructed from error-containing reads, is shown for BCAR (black), PacRAT (red), alignparse (blue), and fgbio (green). Except where indicated, reads were simulated at 1 kb length, with 10 reads per barcode for 1000 barcodes in each condition. (a) Only substitution errors were introduced, not indel errors. The dashed line represents the probability for a given barcode that at least half of the reads have the correct base at all positions, and serves as an approximate theoretical upper bound on the accuracy of BCAR. (b) As in (a), except substitution and indel errors were each introduced at the indicated rates. (c) Accuracy as a function of the length of the target sequence. Reads were simulated with 0.1% indel and substitution rates (dashed lines) or 1% indel and substitution rates (solid lines). (d) Accuracy as a function of the number of reads per barcode. Reads were simulated with 0.1% indel and substitution rates (dashed lines) or 1% indel and substitution rates (solid lines).

We tested BCAR, fgbio, PacRAT, and alignparse for perfect reconstruction of barcoded sequences from simulated reads. We find that BCAR is more accurate than PacRAT or alignparse at moderate error rates (Fig. 5a), and handles indels much better than fgbio (Fig. 5b). PacRAT, alignparse, and fgbio all begin to fail with <1% errors, when most reads will have one or a few errors. BCAR, by contrast, remains highly accurate even when each read has dozens of errors. BCAR begins to fail when sequences are likely to have positions where fewer than half the reads have the correct base call (dashed line, Fig. 5a and b). Although BCAR can theoretically make a correct base call from a plurality of reads, in practice, positions without a majority base call are typically low-confidence and converted to “N.” Therefore, the probability that the correct base call is the majority at all positions represents a soft theoretical bound on BCAR’s accuracy as a function of error rate. BCAR conforms very tightly to this bound when all errors are substitution errors (Fig. 5a), and underperforms it slightly when errors can be substitutions or indels (Fig. 5b), likely due to occasional alignment errors. BCAR is also less sensitive to read length than other methods (Fig. 5c) and can achieve high accuracy with fewer reads per barcode (Fig. 5d).

## 4 Discussion

Accounting for and correcting sequencing errors is a critical part of many sequencing applications. Here, we have described the development of BCAR, a fast and general tool for constructing accurate barcode-sequence maps, even in the presence of indel errors. BCAR accomplishes this by aligning the reads associated with each barcode, using a novel aligner purpose-built for handling sequencing reads. This approach mitigates some of the downsides of using existing multiple sequence aligners.

1. Multiple sequence aligners typically compare genuinely different sequences, with no notion of uncertainty about base identity. In contrast, BCAR uses quality scores to combine evidence across multiple reads, and uses a match/mismatch scoring system based on similarity to compare ambiguous positions.
2. Multiple sequence alignment is typically predicated on an assumption of phylogeny, which does not apply to sequencing reads, and utilizes a guide tree to limit propagation of alignment errors. BCAR aligns reads in order of similarity to an unaligned consensus, which serves a similar function to building a guide tree, but is more appropriate for handling sequencing errors.
3. Multiple sequence alignment is rarely applied to the scale of sequencing datasets, which can contain millions to billions of reads. BCAR is sufficiently fast and memory efficient to handle very large datasets, with sub-millisecond per-read processing time for typical read lengths.

BCAR has some limitations. First, BCAR is designed for individual reads, as opposed to paired reads or sets of reads. Second, BCAR assumes random sequencing errors and, while it flags high-frequency minor alleles, it is not equipped to detect complex structure within a set of clustered reads. Third, BCAR’s accuracy can be slightly reduced with very many reads per barcode (>1000), probably due to the accumulation of rare alignment errors.

That said, we emphasize the flexibility of BCAR. BCAR is platform agnostic rather than being designed specifically for a particular error profile or read length. Furthermore, BCAR inputs and outputs fastq files rather than relying on intermediate file formats or extensive preprocessing. BCAR does not require a reference sequence, meaning it can be applied to many experimental designs. Lastly, BCAR does not impose filtering thresholds on quality scores or read counts, but rather incorporates all available evidence in a Bayesian framework. The user can then choose how to filter their data after BCAR processing. We believe that the combination of speed and flexibility will make BCAR broadly applicable to a wide variety of sequencing datasets.

## Supplementary Material

btag557_Supplementary_Data

## Data Availability

A small subset of sequencing reads is provided in the BCAR repository at https://github.com/dry-brews/BCAR for the purpose of testing. Full raw sequencing datasets are available upon reasonable request.
