# Supplementary material for: BCAR: a fast and indel-tolerant barcode-sequence mapper
Source: Bioinformatics. 2026 Jul 25;42(8):btag557. doi: 10.1093/bioinformatics/btag557 (PMC13433251; doi:10.1093/bioinformatics/btag557)
Supplement: Supplementary file 1 [file btag557_supplementary_data.pdf]

1 Practical advice for running BCAR

The most up-to-date information on BCAR can be found at `https://github.com/dry-brews/BCAR`.

1.1 Pre-processing and post-processing reads

BCAR is designed to be used with other sequence processing tools. A proposed analysis pipeline is presented below. Anecdotally, we find that BCAR performs best after any pre-processing steps have already been performed (e.g., read pairing and trimming).

In order for BCAR to work, it must identify the part of your read that corresponds to the barcode. You can achieve this either by giving BCAR sequence context that flanks your barcode, or by telling it what positions your barcode occurs at. If you are using a position-identified barcode, then it is important that the barcode occurs near the start of the read. Otherwise, any indels occurring prior to the barcode will cause the barcode to be read improperly. In this case, you will want to use tools like Cutadapt [Martin, 2011] or seqtk (`https://github.com/lh3/seqtk`) to trim your reads and place the barcode near the front. If you are using a context-identified barcode, this is not necessary.

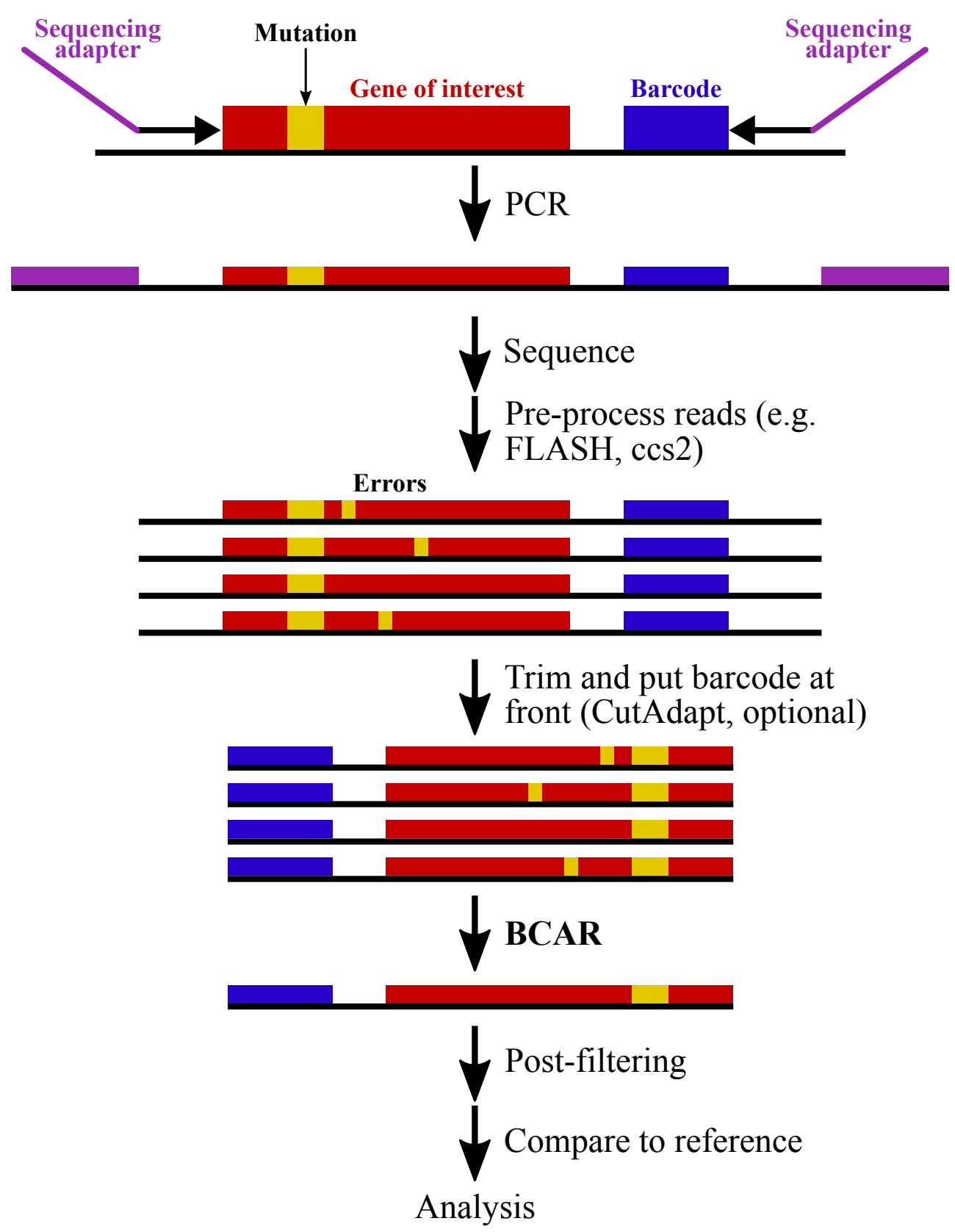

Figure S1: An example workflow that incorporates BCAR with other sequence processing tools to build a barcode-sequence map.

BCAR returns a fastq file with information about the barcode in the header, including the number of reads that contributed to it. You may wish to post-filter your data by requiring a certain number of reads contributed to each barcode, or by thresholding the minimum or average quality score of the consensus sequence.

1.2 Very long reads (>100kb)

If you have very long reads (>100kb), pay special attention to the `--max-len` flag. If you have even a single read that is longer than `--max-len`, it may cause unexpected behavior. You should either pre-trim your reads to a maximum length or determine the longest read length and set `--max-len` slightly higher than that value. For reads of this length, you are likely to run into memory constraints. Memory is shared across all active threads, so if you set fewer threads, more memory will be available to each. Because BCAR uses a memory-efficient banded-alignment strategy, but defaults back to a global alignment when the banded alignment fails, memory usage is also higher when your true indel rate is higher.

1.3 Many substitution errors, few indels

BCAR defaults to a gap penalty of -1.0, which is appropriate when indel errors and substitution errors are approximately equally abundant. On simulated reads, BCAR is quite accurate with this gap score across a broad range of indel and substitution errors. However, if you have very frequent substitution errors (>1%) and quite infrequent indel errors (<0.1%), then using a more severe gap penalty may improve accuracy. A gap penalty of -3.0 is probably appropriate for many such datasets. You can also run BCAR in `--no-alignment` mode, in which case no alignment is performed and speed is increased.

2 Detailed description of algorithm

BCAR performs three passes over a sequencing dataset. In the first pass, the barcodes are identified, allowing them to be efficiently clustered. In the second pass, chunks of reads are sorted based on their barcode cluster identity and the sorted chunks are printed to a set of temporary files. In the third pass, the temporary files are opened in parallel and all of the reads associated with a single barcode cluster are handed off for alignment and merging. This third pass is the only portion that is parallelized across CPU threads.

2.1 Clustering barcodes

Barcodes are first extracted from the raw reads and hashed into a hash table that records their counts in the dataset. Every unique barcode is then given an index and initialized as a cluster. Starting with the most abundant barcode, the possible neighboring barcodes are enumerated based on the user-specified maximum number of indels and substitutions. The barcode cluster in question then absorbs any smaller neighboring clusters that have not yet been through this process. This process continues in sorted order from largest-to-smallest cluster until all barcodes have been considered. To avoid over-merging, BCAR flags when any single cluster contains >0.1% of the total reads if any member is further than `--max-mismatches` away from the centroid.

2.2 Sorting reads

In the second pass over the data, BCAR knows which cluster each barcode belongs to. BCAR loads the reads in chunks, sorts each chunk on the basis of its barcode cluster identity using Quicksort [Hoare, 1962], and prints the sorted chunk to a temporary file. The chunk size can be specified by the user based on hardware memory constraints, and defaults to 4 GB. If more than 240 temporary files are generated (∼1TB of data), then the temporary files are merged hierarchically in batches of 240.

The rest of this section pertains to the third, most computationally intensive pass over the data, where reads are merged into consensus sequences.

2.3 Adjusted quality scores

Raw reads consist of a string of base calls, typically A, C, G, T, or N, and a string of ASCII-encoded quality scores (also called Phred scores), where each score represents:

$$Q_{phred} = -10 * \log_{10}(p_{error}) \tag{1}$$

where p_{error} is the probability that the actual base is different from the base call. Q-scores are typically scaled 1–40, though they can be higher in principle. When generating a consensus sequence, we will want to combine evidence from multiple reads. Q_{phred} scores are not on an additive scale, e.g., the evidence provided by two $Q_{phred} = 20$ base calls is not equal to the evidence provided by one $Q_{phred} = 40$ base call. We define an adjusted quality score, Q_{adj}, which represents one-tenth the evidence conferred by a single base call with $Q_{phred} = 10$. That is:

$$p_{error} = \frac{(1/10)^{Q_{adj}/10}}{(1/10)^{Q_{adj}/10} + (9/10)^{Q_{adj}/10}} \tag{2}$$

We can convert between this score and conventional Q_{phred} scores by setting the two expressions for p_{error} equal to each other:

$$10^{-Q_{phred}/10} = \frac{(1/10)^{Q_{adj}/10}}{(1/10)^{Q_{adj}/10} + (9/10)^{Q_{adj}/10}} \tag{3}$$

$$10^{-Q_{phred}/10} = \frac{(1/10)^{Q_{adj}/10}}{(1/10)^{Q_{adj}/10} + 9^{Q_{adj}/10} * (1/10)^{Q_{adj}/10}} * \frac{10^{Q_{adj}/10}}{10^{Q_{adj}/10}} \tag{4}$$

$$10^{-Q_{phred}/10} = \frac{1}{1 + 9^{Q_{adj}/10}} \tag{5}$$

$$Q_{adj} = \frac{10}{\log_{10}(9)} \log_{10}\left(10^{Q_{phred}/10} - 1\right) \tag{6}$$

Q_{adj} scores are on an additive scale, simplifying combining evidence from multiple reads. Furthermore, they fit naturally into a Bayesian framework for estimating base call confidence. For the 1–40 scale of Q_{phred} scores, Q_{adj} scores range from 0–42. BCAR considers all "N" base calls and all base calls with $Q_{phred} \leq 3$ to contain zero evidence.

It is a quirk of Phred scores that low scores correspond to very high error probabilities, such that a low enough Phred score actually indicates evidence against the putative base call. For instance, $Q_{phred} = 1$ indicates a 79% probability that the base call is incorrect. We define $Q_{adj} = 0$ for $Q_{phred} \leq 3$, rather than converting them to negative values, on the intuition that people generally do not intend to indicate negative evidence with a low score, but merely the lack of strong evidence.

In summary, we use the following conversion:

Q_{phred}	Q_{adj}	Q_{phred}	Q_{adj}	Q_{phred}	Q_{adj}	Q_{phred}	Q_{adj}	Q_{phred}	Q_{adj}
0	0	9	9	18	19	27	28	36	38
1	0	10	10	19	20	28	29	37	39
2	0	11	11	20	21	29	30	38	40
3	0	12	12	21	22	30	31	39	41
4	2	13	13	22	23	31	32	40	42
5	4	14	14	23	24	32	34	>40	42
6	5	15	16	24	25	33	35		
7	6	16	17	25	26	34	36		
8	8	17	18	26	27	35	37		

2.4 Merging reads into an alignment

Upon observing a read, BCAR converts the read into an array, where the rows represent [A,C,G,T,-] and the columns represent positions. At each position, the cell representing the base call for the read is filled with the Q_{adj} score and the other cells are filled with zero. The fifth row, "-", represents a gap character that does not appear in the raw reads but may be used during alignment.

Prior to alignment, BCAR builds an unaligned consensus of the raw reads by adding the read matrices element-wise. Then, the reads are sorted according to their similarity to the unaligned consensus, with the most similar reads being merged first to limit propagation of alignment errors. The reads are iteratively pairwise aligned using the Needleman-Wunsch algorithm [Needleman and Wunsch, 1970], adapted for ambiguous matches. While the Needleman-Wunsch algorithm typically uses fixed "match" and "mismatch"

scores (commonly, 1 and -1), BCAR must compare positions where there is ambiguity about the base call. For the two vectors representing the positions being compared between the read array and the consensus array, the match score is taken to be the cosine similarity between the vectors, linearly rescaled from $[0, 1]$ to $[-1, 1]$. These values, along with a user-specified gap penalty, are used to fill a dynamic programming array in the usual way (but see section 2.5). The traceback from this array directs which positions to merge between the two sequences. For the aligned positions, the vectors representing the positions are simply added element-wise. When a gap is proposed, the base calls from the sequence that don't support a gap are maintained, and the base calls from the sequence that do support a gap are summed and added to the gap field ("-") of the new alignment. Iterative pairwise alignment continues until all reads are accounted for.

2.5 Banded alignment

To optimize speed and memory usage, BCAR uses a banded alignment strategy [Hirschberg, 1975], where the dynamic programming array is only filled within a diagonal band of fixed width. The optimal path within the band is not guaranteed to be an optimal global alignment, but one can check whether it is optimal by comparing it to the highest possible score that would leave the band [Gibrat, 2018]. The width of the band therefore balances between speed and the likelihood of finding an optimal alignment, and the best choice of band width is a function of the indel rate. BCAR works by first choosing a band width of $\sqrt{L/25}$, where L is the longer of the read length or the consensus length, based on guessing an indel rate of ∼1% [Gibrat, 2018]. If the resulting alignment is found to be optimal, it is accepted. Otherwise, the band is progressively widened until an optimal alignment is found, falling back to a full global alignment if necessary.

2.6 Generating a consensus

Each position in the final alignment is represented by a vector of evidence for each base call (including gaps). Positions with a plurality of evidence for a gap are skipped and do not contribute to the consensus read. For other positions, the base call with the most evidence is proposed as the true base. Evidence for all other bases (excluding gaps) is summed as evidence against the proposed base call. Bayes' theorem is applied to calculate a Q_{phred} score for the proposed base call as follows. For a base call b in $B \in [A, C, T, G, -]$

$$\log_{10}(p_b) = \frac{Q_b}{10} * \log_{10}(\frac{9}{10}) + \sum_{i \neq b}^{B} \left(\frac{Q_i}{10} * \log_{10}(\frac{1}{10}) \right) \tag{7}$$

So, p_b represents the non-normalized probability of base b being the true base at each position. The probabilities of the bases that are not proposed as the consensus base can be summed and normalized to produce a total probability of error, which can be converted to a Q-score.

$$Q_{phred} = -10 * \log_{10} \left(\frac{\sum_{i \neq max(B)}^{B} p_i}{\sum_{i}^{B} p_i} \right) \tag{8}$$

By convention, BCAR caps the quality scores $Q_{phred} \leq 40$, and for Q_{phred} values of 1–3, the base call is converted to an "N".

3 Simulating reads

Barcoded reads were simulated for the purpose of testing accuracy of error correction. For each barcode, a wild-type sequence was generated by randomly sampling L bases with equal probability. The first 15 base pairs were designated as the barcode. Then, for each barcode, R reads were generated by copying the wild-type sequence at each position while allowing for the possibility of errors. First, an indel was allowed with probability μ_i, equally split between (single-base) insertions and deletions. Insertions were given a random base. Then, if no indel occurred, a substitution was allowed with probability μ_m, equally split between the three mutant bases. When the true wild-type base was copied, a quality score was sampled from a normal distribution, $N(\mu = 30, \sigma = 10)$, truncated to [1,40]. When a miscall occurred, a quality score was sampled from $N(\mu = 20, \sigma = 10)$, truncated to [1,40].

4 Experimental data collection

The test data come from two mutational scanning libraries. For the "AVITI" library, DNA minipreps were prepared for sequencing using a 2-round PCR strategy, which adds phased sequencing adapters on both sides, library-specific dual indices, and TruSeq-style Illumina adapters. The samples were then sent to the University of Chicago genomics core for paired-end 300-cycle reads (PE300) on the Element AVITI. For the "PacBio" library, DNA minipreps were linearized by a restriction enzyme which cuts on the plasmid backbone. The linearized product was sent to SeqCenter LLC for sequencing on the PacBio Revio system.

4.1 Pre-analysis read processing

For both libraries, the raw reads were trimmed using Cutadapt to remove the adapter sequences and leave only the sequence between the start codon and the last base of the barcode [Martin, 2011]. Reads without adapters were discarded. PacBio reads can occur in either orientation, so Cutadapt was run in each orientation and the resulting trimmed sequences were put into the same orientation and combined. The AVITI dataset consists of 385 million reads covering 19 million barcodes, with $\sim$20 reads per barcode, on average. The PacBio dataset consists of 7.7 million reads covering 740,000 barcodes, with $\sim$10 reads per barcode, on average.

5 Analysis of AVITI library

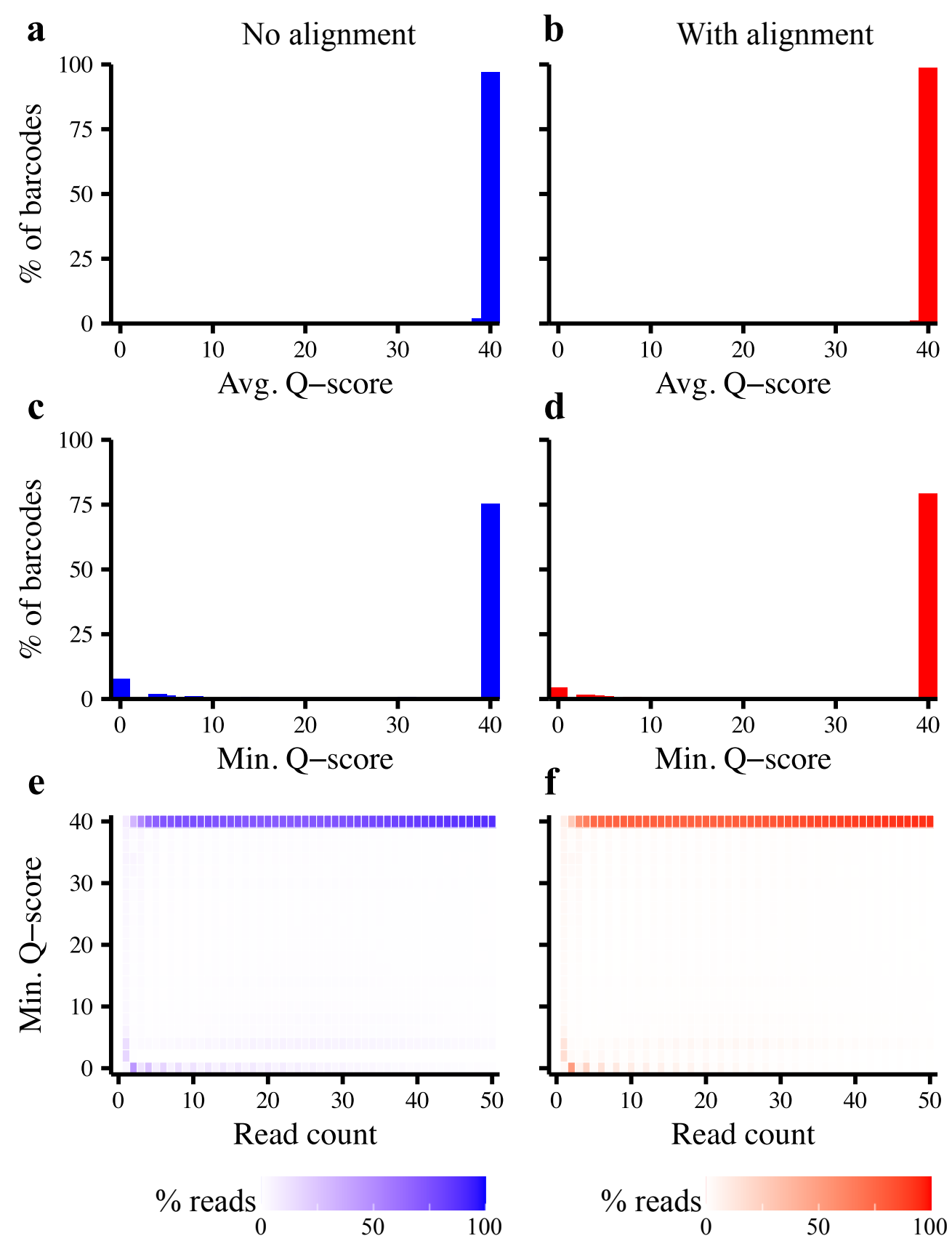

Figure S2: **(a-b)** Average Q-score distribution of consensus reads from BCAR, run without (a) and with (b) alignment. **(c-d)** Minimum Q-scores of consensus reads from BCAR, run without (c) and with (d) alignment. **(e-f)** Minimum Q-score as a function of the number of reads per barcode, for BCAR run without (e) and with (f) alignment.

The AVITI library, described in "Application to experimental data", was analyzed by BCAR with and without alignment (Fig. S2). Using alignment, BCAR recovers some consensus reads that would be of very low quality without alignment (Fig. S2c-d). However, the effect is less pronounced than in the PacBio dataset. Although we observed the AVITI and PacBio datasets to contain similar per-base indel rates, the PacBio reads, being much longer, had many more indels per read. Additionally, the AVITI dataset has about twice as many reads per barcode. We observe a dramatically higher indel rate in our data compared to previously published estimates [Liu et al., 2024], and it is possible that this indicates library preparation artifacts such as PCR strand-exchange, rather than true sequencing errors. BCAR may be less able to correct these artifacts, as it is designed to correct random, rather than systematic, errors.

6 Speed

For clustering barcodes, the slow step is enumerating the possible neighbors for each barcode, which is exponential in time with respect to the number of allowed errors. For this reason, it is recommended to allow no more than ~ 3 barcode errors, regardless of barcode length. Sorting the reads is $O(N * \log(N))$, and is generally IO-limited. The speed of merging reads is more complicated, depending on the length of reads and the frequency of indels. Because of the banded alignment strategy, in the best case, merging reads is $O(N * L)$, whereas in the worst case, it is $O(N * L^2)$. Average memory usage mirrors speed, but peak memory usage should be assumed to be $O(N * L^2)$.

Datasets with many short reads will require more time to cluster and sort, whereas datasets with fewer long reads will require more time to merge. For example, the PacBio dataset with 7.7 million ∼3500 bp reads took only ∼8 CPU-minutes to cluster and sort, but ∼54 CPU-minutes to merge. In contrast, the AVITI dataset with 385 million ∼350 bp reads took 79 CPU-minutes to cluster and sort, but only 71 CPU-minutes to merge. Both tests were performed on 24–thread compute nodes (Intel E5-2680 v4 2.40 GHz).

6.1 Speed benchmarks

We tested the speed of BCAR on simulated reads with varied length and reads per barcode (Fig. S3). All times shown are wall-clock times that include all steps of BCAR. Testing was performed on a 24–thread compute node (Intel E5-2680 v4 2.40 GHz), using all threads for parallel computation. Except where otherwise noted, reads were generated with 10 reads per barcode and a 1kb read length with the first 15 bases designated as the barcode. Time taken per read increases sublinearly for read lengths shorter than ∼400 bp, when clustering and IO dominate, and increases linearly-to-quadratically for longer reads, when Needleman-Wunsch alignment dominates (Fig. S3a). Adding more reads per barcode can make the per-read time longer or shorter (Fig. S3b), depending on the tradeoff between spawning new threads and spending more time on challenging alignments, with low error rates generally getting faster (fewer threads to spawn) and high error rates generally getting slower (challenging alignments). For most reasonable distributions of reads per barcode, users can expect time to scale approximately linearly with number of reads.

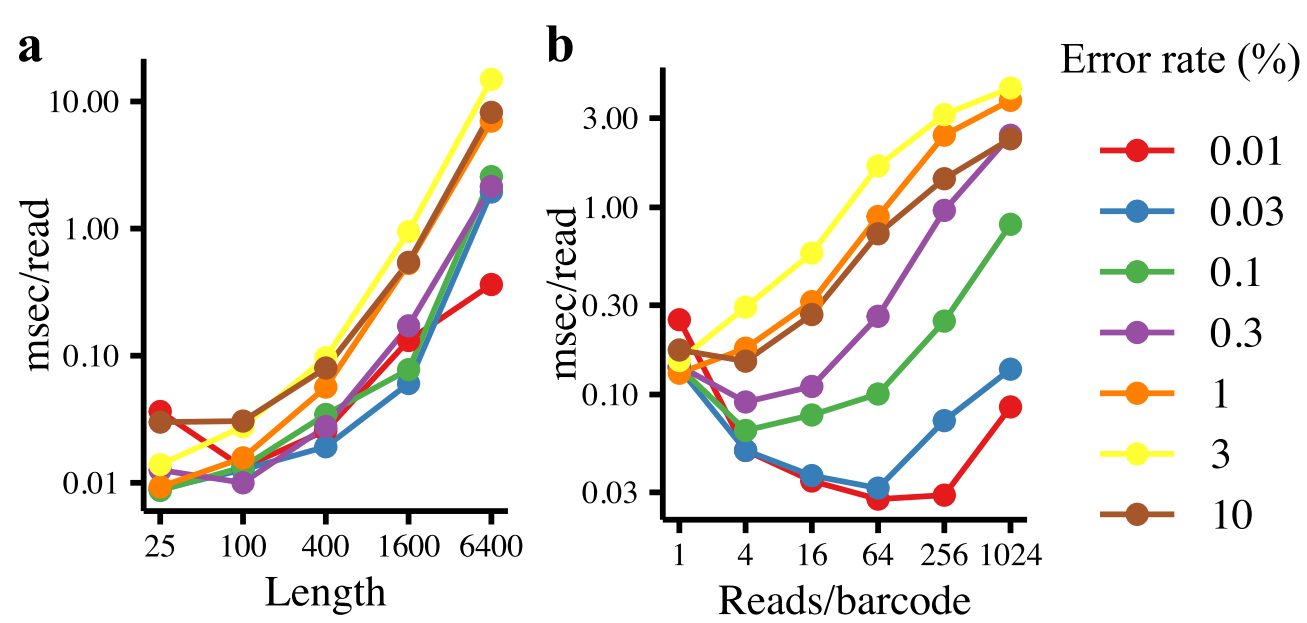

Figure S3: **(a)** Time taken per read as a function of read length. Note the log scale on both axes. **(b)** Time taken per read as a function of the number of reads per barcode. Increasing error rate increases the time taken per read, likely due to the increased probability of needing to widen the banded alignment.

References

Jean-François Gibrat. A short note on dynamic programming in a band. *BMC bioinformatics*, 19(1):226, 2018.

Daniel S. Hirschberg. A linear space algorithm for computing maximal common subsequences. *Communications of the ACM*, 18(6):341–343, 1975.

Charles AR Hoare. Quicksort. *The computer journal*, 5(1):10–16, 1962.

Silvia Liu, Caroline Obert, Yan-Ping Yu, Junhua Zhao, Bao-Guo Ren, Jia-Jun Liu, Kelly Wiseman, Benjamin J Krajacich, Wenjia Wang, Kyle Metcalfe, et al. Utility analyses of aviti sequencing chemistry. *BMC genomics*, 25(1):778, 2024.

Marcel Martin. Cutadapt removes adapter sequences from high-throughput sequencing reads. *EMBnet. journal*, 17(1):10–12, 2011.

Saul B Needleman and Christian D Wunsch. A general method applicable to the search for similarities in the amino acid sequence of two proteins. *Journal of molecular biology*, 48(3):443–453, 1970.
